# Endocardite por Coxiella Burnetii: A Tomografia por Emissão de Pósitrons pode ser uma Alternativa ao Diagnóstico?

**DOI:** 10.36660/abc.20210421

**Published:** 2022-06-06

**Authors:** Marjorie Hayashida Mizuta, Cristhian Espinoza Romero, Santiago Castro Vintimilla, Tatiana de Carvalho Andreucci Torres Leal, Paulo Rogério Soares, Alexandre de Matos Soeiro

**Affiliations:** 1 Universidade de São Paulo Instituto do Coração São Paulo SP Brasil Instituto do Coração – Universidade de São Paulo, São Paulo, SP – Brasil

**Keywords:** Endocardite, Coxiella Burnetti, Febre Q, Proteses Valvulares Cardíacas, Diagnóstico por Imagem, Ecocardiograma Transesofagiana/métodos, Tomografia Computadorizada por Emissão de Pósitrons/métodos, Antibióticos/uso terapêutico

## Introdução

A endocardite infecciosa (EI) por *Coxiella burnetii* representa uma zoonose com raros relatos no Brasil.^1^ Estima-se que a *Coxiella burnetii* seja responsável por até 5% de todos os casos de EI no mundo.^2^ A doença acomete preferencialmente valvopatas e imunocomprometidos.

Diferente das formas clássicas de endocardite aguda e subaguda, o quadro clínico é frustro e, por se tratar de um micro-organismo intracelular obrigatório, as hemoculturas (HMC) são predominantemente negativas, dificultando a suspeita clínica.^1^

Apresentamos um caso clínico raro de endocardite que se manifestou de forma atípica e foi diagnosticado com auxílio da sorologia específica para *Coxiella burnetii* e da tomografia por emissão de pósitrons (PET).

## Relato de caso

**Figura 1:**
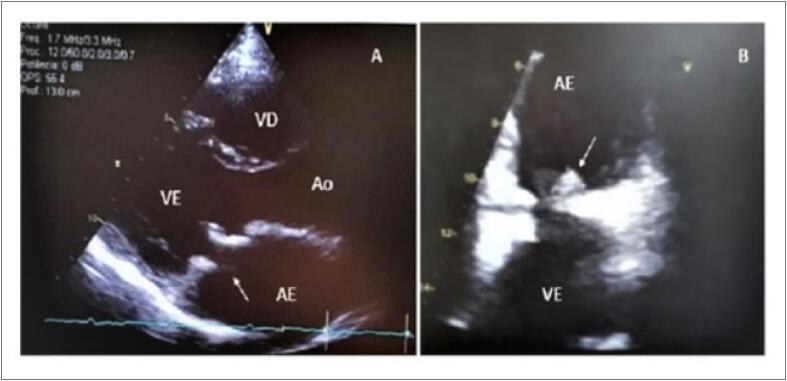
A) ETT 2D em posição paraesternal longitudinal demonstrando prótese mitral com espessamento dos seus folhetos, aspecto de pannus. B) ETE 2D a 60º demonstrando imagem ovalar aderida à face atrial do folheto posterior. VD: ventrículo direito; VE: ventrículo esquerdo: Ao: aorta; AE: átrio esquerdo.

**Figura 2:**
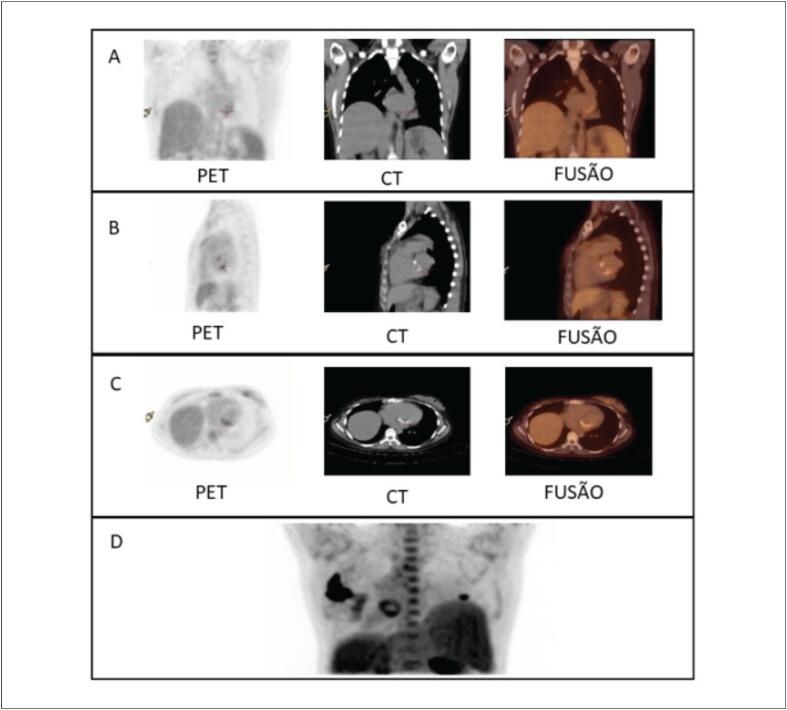
Imagens geradas por PET, tomografia computadorizada (CT) e fusão. PET demonstrou captação difusa de fluordeoxiglicose em topografia valvar mitral nos planos coronal (A), sagital (B), axial (C), e em 3D (D).

Paciente de 25 anos do sexo feminino, natural e procedente de Monte Santo – Bahia, técnica de agropecuária, apresentava antecedente de duas trocas na valva mitral por prótese biológica devido a doença valvar reumática, sendo a última em 2017. Compareceu à unidade de emergência após ser encaminhada do ambulatório de valvopatias por suspeita de EI. Referia que, em julho de 2020, apresentou quadro de lesões eritematosas em membros inferiores e superiores associadas a febre baixa intermitente que se estenderam por 6 meses, com resolução completa do quadro após o uso de cefalexina por 5 dias, em janeiro de 2021. Na admissão hospitalar, em fevereiro de 2021, estava assintomática, não apresentava alterações ao exame físico e trazia um ecocardiograma transtorácico (ETT) realizado ambulatorialmente há 5 dias que mostrava prótese biológica mitral com espessamento dos seus folhetos com aspecto de pannus, não sendo possível descartar vegetação (Figura 1A). Foi solicitado ecocardiograma transesofágico (ETE) para melhor visualização da prótese valvar. O ETE mostrou imagem ovalar, de bordos bem definidos, aderida à face atrial da base do folheto posterior, medindo 11x5 mm podendo corresponder a vegetação ou fio de sutura com fibrina, sem disfunção da prótese mitral (Figura 1B). Dada a hipótese de EI, a paciente foi internada e foram solicitados 3 pares de HMC e exames laboratoriais que mostraram leucócitos de 6720/mm³, velocidade de hemossedimentação de 18 mm, proteína C-reativa de 18 mg/dl, e urina I e perfil hepático normais. Devido à estabilidade clínica, a paciente foi mantida sem antibióticos até o resultado das hemoculturas estar disponível. Como as hemoculturas foram negativas, optou-se por solicitar sorologias para *Coxiella burnetii* e *Bartonella henselae*. A sorologia foi reagente para *Coxiella burnetii* (título >1:1.600), sendo iniciado tratamento no segundo dia de internação com ciprofloxacino 400 mg endovenoso 12/12h por 7 dias associado a doxiciclina 100 mg via oral (VO) de 12/12h e hidroxicloroquina 200 mg VO 8/8h por 18 meses. Complementando a investigação, no terceiro dia de internação foi realizado PET que, em vigência de antibioticoterapia, mostrou atividade inflamatória na região valvar mitral, porém com a possibilidade de processo infeccioso em resolução (Figura 2). A paciente evoluiu com estabilidade hemodinâmica, recebendo alta hospitalar após 8 dias de internação com antibioticoterapia descrita.

## Discussão

A EI por *Coxiella burnetii* representa uma zoonose com ampla distribuição mundial. A forma de transmissão mais comum em humanos é a inalação de aerossóis derivados de secreções orgânicas do gado, durante o parto ou na forma de ingestão de leite cru contaminado.^1^ No caso clínico, como a paciente apresentava risco ocupacional, a possibilidade de infecção por *Coxiella burnetii* era altamente suspeita.

A maioria dos pacientes apresenta sintomas insidiosos de insuficiência cardíaca e sintomas inespecíficos como febre baixa e fadiga. No exame físico pode haver presença de hepatoesplenomegalia e baqueteamento digital.^1^ As manifestações cutâneas são incomuns e podem ser representadas por erupções purpúricas, puntiformes ou maculopapulares, e aparecem comumente na forma aguda da doença.^3^ Como a paciente relatava manifestação cutânea há 7 meses da admissão, na internação hospitalar apresentava, provavelmente, a fase crônica da doença.

A sorologia compõe um dos critérios maiores de Duke para EI por *Coxiella burnetii*. Constitui um marcador diagnóstico da infecção crônica quando apresenta títulos de anticorpos IgG antifase-I >1:800, com elevada sensibilidade e especificidade.^1^

O ETT é capaz de revelar anormalidades em apenas 12% dos casos devido à *presença de vegetações pequenas, nodulares ou planas que passam despercebidas mesmo no ETE.*^*1*^

O PET tem demostrado valor diagnóstico na EI de prótese valvar ou de dispositivo intracardíaco (sensibilidade de 87% e especificidade de 92%). Foi incorporado no algoritmo diagnóstico das diretrizes e não é recomendado em valva nativa nem no pós-operatório precoce.^4^ Existem vários relatos de utilização do PET-CT, como ferramenta diagnóstica na EI por *Coxiella burnetii*, sugerindo que essa técnica pode auxiliar na localização da infecção em pacientes com evidência sorológica de infecção persistente. ^5^

Dessa forma, apresentamos o caso de uma EI com alta mortalidade quando não tratada precocemente. O diagnóstico é dificultado devido ao comportamento crônico da doença. A vegetação é inespecífica ao ecocardiograma e as hemoculturas são negativas.^1^ O PET e a sorologia se destacam nesse cenário, pois um exame ecocardiográfico inconclusivo não exclui o diagnóstico em pacientes com alta suspeição de EI.^6^ No caso clínico, mesmo em vigência de antibioticoterapia e sob a forma crônica da doença, o PET pôde inferir e localizar a infecção, possibilitando um diagnóstico mais preciso e evitando desfechos letais.
