# Supplementary material for: Endocardite por Coxiella Burnetii: A Tomografia por Emissão de Pósitrons pode ser uma Alternativa ao Diagnóstico?
Source: Arq Bras Cardiol. 2022 Jun 6;118(6):1144–6. [Article in Portuguese] doi: 10.36660/abc.20210421 (PMC9345161; doi:10.36660/abc.20210421)
Supplement: Translation

# Translation: Coxiella Burnetii Endocarditis: Can Positron Emission Tomography be an Alternative to Diagnosis?

**Authors:** Marjorie Hayashida Mizuta, Cristhian Espinoza Romero, Santiago Castro Vintimilla, Tatiana de Carvalho Andreucci Torres Leal, Paulo Rogério Soares, Alexandre de Matos Soeiro

**Affiliations:** 1 São Paulo SP Brazil Instituto do Coração – Universidade de São Paulo, São Paulo, SP – Brazil

**Keywords:** Endocarditis, Coxiella Burnetti, Q Fever, Heart Valve Prosthesis, Echocardiography, Transesophageal/methods, Positron Emission Tomography Computed Tomography/methods, Antibiotics/therapeutic use

## Introduction

*Coxiella burnetii* infective endocarditis (IE) represents a rarely reported zoonosis in Brazil.^1^ It is estimated that *Coxiella burnetii* is responsible for up to 5% of all IE cases worldwide.^2^ The disease affects mostly valvulopathy patients and immunocompromised subjects.

Different from the classical acute and sub-acute endocarditis, the clinical picture is frustrating, and, because this is an obligate intracellular microorganism, hemocultures (HMC) are predominantly negative, which makes the clinical suspicion more difficult.^1^

This paper presents a rare endocarditis clinical case that manifested in an atypical manner, and was diagnosed with the help of specific *Coxiella burnetii* serology and of the positron emission tomography (PET).

## Case report

A 25-year-old female patient, was born in and is a resident of Monte Santo, Bahia and who is a farming technician, had a history of two mitral valve replacements with biological prosthesis due to rheumatic valve disease, with the most recent occurrence in 2017. She was referred to the emergency unit by the valvulopathy clinic with suspected IE. The patient reported that, in July 2020, she exhibited erythematous lesions in the lower and upper limbs, associated with intermittent low-grade fever, which persisted for 6 months. The picture was fully solved with the use of cephalexin for 5 days, in January 2021. Upon hospital admission, in February 2021, the patient was asymptomatic, exhibited no alterations in the physical examination, and provided a transthoracic echocardiogram (TTE) performed at the clinic 5 days before showing a mitral biological prosthesis whose leaflets exhibited pannus formation, with the possibility of vegetation not excluded (Figure 1A). A transesophageal echocardiogram (TEE) was scheduled to better view the valvular prosthesis. The TEE showed an oval shape, with well-defined borders, which adhered to the posterior leaflet base atrial face, measuring 11x5 mm, and which may correspond to vegetation or suture thread with fibrin, with no mitral prosthesis dysfunction (Figure 1B). Given the IE hypothesis, the patient was admitted to the hospitals and 3 pairs of HMC and laboratory tests were conducted showing 6,720/mm³ white blood cells, an erythrocyte sedimentation rate at 18 mm, C-reactive protein at 18mg/dl, and normal urine I and liver function tests. Due to the clinical stability, the patient was kept off antibiotics until the hemoculture results were available. As the hemocultures were negative, *Coxiella burnetii* and *Bartonella henselae* serology tests were conducted. Serology was reactive to *Coxiella burnetii* (titer >1:1,600), and treatment was started on the day after hospital admission, combining intravenous 400 mg ciprofloxacin every 12 hours for 7 days, 100 mg oral delivery (OD) doxycycline every 12 hours, and 200 mg OD hydroxychloroquine every 8 hours for 18 months. To complement the investigation, on the third day of hospital stay, a PET was conducted, which, with the use of antibiotic therapy, showed inflammatory activity in the mitral valve area; however, it was deemed possible that the infective process was being resolved (Figure 2). The patient progressed with hemodynamic stability and was discharged after 8 days of hospital stay with the described antibiotic therapy.

**Figure 1:** A) 2D TTE in longitudinal parasternal position showing mitral prosthesis with thickened leaflets and pannus aspect. B) 2D TEE at 60º showing an oval shape adhered to the posterior leaflet atrial face. RV: right ventricle; LV: left ventricle; AO: aorta; LA: left atrium.

**Figure 2:** PET, computed tomography (CT), and fusion generated images. PET demonstrated diffuse fluorodeoxyglucose uptake in mitral valve topography in the coronal (A), sagittal (B), and axial (C) planes, and in 3D (D).

## Discussion

*Coxiella burnetii* IE represents a worldwide spread zoonosis. The most common means of transmission in human beings is the inhalation of aerosols derived from cattle organic secretion, during birth or by ingesting contaminated raw milk.^1^ In the clinical case, as the patient was under occupational risk, the possibility of *Coxiella burnetii* infection was highly suspected.

Most patients exhibit insidious symptoms of heart failure, and unspecified symptoms, such as low-grade fever and fatigue. The physical examination may detect the presence of hepatosplenomegaly and digital clubbing.^1^ Skin manifestations are uncommon and may be represented by purpuric, punctiform, or maculopapular eruptions, and are commonly present in the acute form of the disease.^3^ As the patient reported a skin manifestation 7 months prior to admission, during the hospital stay period, the patient was probably in the chronic phase of the disease.

Serology is one of Duke’s major criteria for *Coxiella burnetii* IE. It constitutes a diagnostic marker of chronic infection when it exhibits antiphase I IgG antibody titers >1:800, with high sensitivity and specificity.^1^

The TTE is capable of revealing abnormalities in only 12% of the cases, due to the presence of small, nodular, or flat vegetations that go unnoticed even in the TTE.^1^

The PET has demonstrated a diagnostic value in valve prosthesis or intracardiac device IE (87% sensitivity and 92% specificity). It was incorporated in the guideline diagnosis algorithm and is not recommended for native vales or early postoperative stages.^4^ There are several reports of PET-CT being used as a diagnostic tool in *Coxiella burnetii* IE, suggesting that this technique may help in locating the infection in patients with serological evidence of persistent infection.^5^

Therefore, this paper presents an IE case with high mortality if not treated early. The diagnosis is difficult due to the chronic behavior of the disease. The vegetation is unspecified to the echocardiogram and hemocultures are negative.^1^ The PET and serology stand out in this scenario, considering that a non-conclusive echocardiogram does not exclude the diagnosis in patients with highly suspected IT.^6^ In the clinical case, even during antibiotic therapy and in the chronic form of the disease, the PET was able to infer and locate the infection, allowing for a more precise diagnosis and avoiding lethal outcomes.
